# Clinical development innovation in rare diseases: overcoming barriers to successful delivery of a randomised clinical trial in alkaptonuria—a mini-review

**DOI:** 10.1186/s13023-022-02606-0

**Published:** 2023-01-04

**Authors:** L. R. Ranganath, Nick Sireau

**Affiliations:** 1 Department of Clinical Biochemistry and Metabolic Medicine, Royal Liverpool University Hospital, Prescot Street, Liverpool, L7 8XP UK; 2 Department of Musculoskeletal Biology, University of Liverpool, Liverpool, L7 8TX UK; 3 The Alkaptonuria Society, 66 Devonshire Road, Cambridge, CB1 2BL UK

**Keywords:** Alkaptonuria, Nitisinone, Homogentisic acid, SONIA 2, DevelopAKUre, Clinical trial, Barriers

## Abstract

Alkaptonuria is a rare inherited disorder for which there was no disease-modifying treatment. In order to develop a successful approved therapy of AKU multiple barriers had to be overcome. These included activities before the conduct of the study including deciding on the drug therapy, the dose of the drug to be used, clarify the nature of the disease, develop outcome measures likely to yield a positive outcome, have a strategy to ensure appropriate patient participation through identification, build a consortium of investigators, obtain regulatory approval for proposed investigation plan and secure funding. Significant barriers were overcome during the conduct of the multicentre study to ensure harmonisation. Mechanisms were put in place to recruit and retain patients in the study. Barriers to patient access following completion of the study and regulatory approval were resolved.

## Introduction

Alkaptonuria (AKU) (OMIM#203500) is a serious, rare autosomal recessive disorder due to deficiency of homogentisate dioxygenase with resultant accumulation of homogentisic acid (HGA), occurring with a worldwide frequency of 1 in 250,000 [1]. The accumulation of HGA results in progressively conversion and deposition to a melanin-like HGA-pigment in connective tissue, causing tissues to become rigid and brittle, and prone to degradation. This disease-specific pigmentary process is called ochronosis [2–4], which causes multiple systems to be involved resulting in varying phenotypes, characterised by severe premature spondyloarthritis, lithiasis, cardiac valve disease, fractures, muscle and tendon ruptures, and osteopenia [5, 6]. The disease is slowly progressing with a pre-symptomatic phase, apart from dark urine, until clinical signs and symptoms appear, usually when the patients are in their late twenties [7]. For our research, the multifaceted aspects of the disease required multiple assessments so that the most appropriate endpoints could be defined. The result was the need to collect large amounts of data, meaning that studies that needed to be performed were complex. This also required compliance and willingness on the part of patients to ensure a successful study. At the same time, these aspects also require a large sample size for statistical power of the study despite the rarity of AKU, a significant challenge.

We were fortunate that a potential drug called nitisinone (NIT) was available to test in AKU. Until 1998 there was a lack of HGA-lowering disease-modifying therapy [8] when it was suggested that NIT, already in use as treatment of hereditary tyrosinaemia type 1 (OMIM 276700) [9], could also decrease HGA [10]. NIT inhibits the enzyme *p*-hydroxyphenyl-pyruvate dioxygenase (HPPD) (EC:1.13.11.27), thereby decreasing the accumulation of HGA. [10, 11]. Ochronotic pigment develops from HGA; therefore, a decrease in HGA could decrease pigment, and consequently the morbidity of AKU. Initial studies employed an oral dose of 2 mg daily even though a full dose–response profile was never undertaken, and adopted for the first NIT interventional study [11]; 20 NIT-treated patients were compared with 20 controls, employing lateral rotation of the hip as the primary outcome, with the study reporting inconclusive. As a result, a consortium of European investigators came together to conduct a European Commission-funded study called the Suitability of Nitisinone in Alkaptonuria 2 (SONIA 2), a 4-year phase 3 NIT outcomes study in AKU. Our experiences in identifying and overcoming barriers in this SONIA 2 study, before starting the study during the preparatory phase, during the conduct of the study and then post-study activities to enable regulatory approval of the first disease-modifying therapy for adults with AKU is described in greater detail here.

## Pre-study preparations

The rarity and the lack of serious morbidity in early years has hindered the advances in knowledge needed to develop effective therapies; while case reports of single or few cases continue to appear in the medical literature, good studies describing significant numbers of AKU cases remain scant. The activities of the Natural History Study at the National Institutes of Health in the USA, as well as the United Kingdom National Alkaptonuria Centre have helped update knowledge about AKU. The pioneering suggestion by the NIH that a drug already treating another fatal inherited disorder, hereditary tyrosinaemia 1 or HT-1, led to initial development of an enzyme inhibitor NIT with the outcomes study however proving inconclusive and not leading to FDA approval for use in AKU [8–11]. This made it harder to convince the marketing authorisation holder of nitisinone (Swedish Orphan Biovitrum, Sobi) to support a new clinical NIT trial. We realised that the barriers to successful clinical NIT trial were low numbers of recruitable subjects, incomplete understanding of the nature of AKU and lack of an appropriately representative outcome measure. These barriers were addressed and resolved by carrying out a population identification study which enabled identification of 75 UK patients and 626 outside the UK [12]. A natural history study re-emphasises the delayed slow inexorable progression of AKU so that the outcomes study was longer [13]. The natural history study recognised the multisystem nature of AKU and developed a composite score describing the burden of AKU disease in a patient that could serve as an outcome measure instead of a single variable disease feature [14, 15]. Further the NIT license holder Sobi obtained regulatory approval for use in HT-1 in 2002 in the USA and 2005 in Europe and UK [16]; when the clinical studies were being planned in 2011 it became clear that the 12-year market exclusivity for NIT in HT-1 would run out in during the planned AKU repurposed NIT outcomes study making it difficult to convince Sobi to support the study by providing expertise and NIT [17]. Drug repurposing is more economic that starting from scratch for orphan diseases. Obtaining funding to study and develop effective treatments for rare disease is challenging with over 7000 competing rare disease; we laud European strategy on rare disease focus which offers funding hope for millions of rare disease sufferers. The European Commission funded DevelopAKure, the series of studies including the 4-year randomised control trial, through it’s Framework 7 programme and provided €6,000,000; Sobi as the pharmaceutical partner provided NIT free of charge in DevelopAKUre. A barrier to success in new clinical therapy development can be overcome by engaging with the regulatory authorities to develop an acceptable study design in consultation with these agencies to have seamless regulatory approval following conclusion of study; we engaged with European Medicines Agency and they understood the complexities of the development and guided us to use a realistic metabolic end point while also expecting us to show trends in clinical benefit (Table 1). As a result, the European Commission Framework Programme 7 funded a programme of studies entitled DevelopAKUre which commenced in November 2012.

**Table 1:** Barriers and solutions during the preparatory stage

Barrier	How addressed
Very rare genetic disease	Transnational collaboration
Lack of knowledge	Carry out natural history study, establishment of single centres of expertise
Low patient numbers	Intensive identification of patients in Europe and beyond
Previous failed study	Understoodd reasons and addressed these
Lack of reliable outcome measure	Developed multisystem assessment tool AKUSSI
Availability of drug	Repurposing nitisinone already in use for another condition (HT-1)
Lack of knowledge of dose of study drug	SONIA 1 dose–response study carried out
Robust pharmaceutical partner	Despite loss of market exclusivity and availability of generics, company ethos and persuasion was successful in Sobi joining the consortium
Regulatory acceptance of clinical development	EMA engagement for advice on planned study
Building consortium	Strong record of working well already and reliable personal contacts
Funding	Grant application to the EC; nitisinone provided free of change by Sobi

## Peri-study considerations

**Fig. 1:**
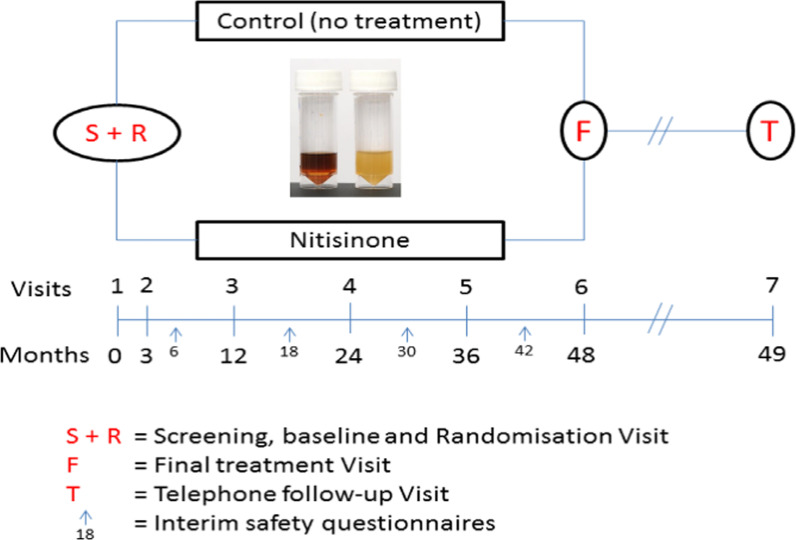
The SONIA 2 study design. 69 patients received nitisinone while 69 controls did not after screening and randomisation at baseline visit. Following the baseline visit, patients visited study sites at month 3 and then 12, 24, 36 and 48 months. Safety questionnaires were administered at 6, 18, 30 and 42 months. The inset figure of dark urine and less dark comparator indicated that patients could not be blinded to the study due to nitisinone therapy clearing the dark urine. All assessors of data were blinded to the treatment

The DevelopAKUre programme was a 5.5-year duration to deliver on a dose–response as well as an outcomes study called Suitability of Nitisinone in Alkaptonuria 1 and 2 respectively (SONIA 1 and SONIA 2). SONIA 2 started only after SONIA 1 confirmed the final dose that was used in SONIA 2 after 1.5 years from start of study, requiring frequent notifications to the EC while obtaining approval to make changes to study logistics in order to finish as close to agreed timelines as possible despite which the study overran by 9 months. Despite the UK having identified 76 AKU patients, they could not be recruited to SONIA 2 due to ethical dilemma of these patients being randomised to the control no-NIT arm when they were eligible to receive NIT for free in the UK NAC; this necessitated patient recruitment especially for the UK site from Europe resulting in barriers such as overseas travel for disabled patients, language barriers requiring use of interpreters as well as carefully planned safety rescue for patients if they suffered an adverse event. SONIA 2 was challenging due to a number of factors including lengthy multisystem assessments lasting from Monday to Friday of each study site visits. The SONIA 2 study sites were Liverpool (UK), Piešťany (Slovakia) and Paris (France) (Fig. 1). The study procedures and processes were harmonised to minimise variability in procedures such as serum sample acidification, ear cartilage biopsy and photographs of eyes and ears for HGA-pigment to name a few; Liverpool was an adult general hospital, Piešťany was a specialist national rheumatology centre while France was a paediatric metabolic centre. Site initiation visits before commencement was used to minimise variability. We had effective patient societies assisting and supporting patients in Liverpool and Paris but had difficulty in Piešťany. We recruited 139 out of the projected 140 patients in SONIA 2 over 9 months thanks to the activism of our patient societies; this was helped by having a weekly recruitment telemeetings. The recruitment telemeetings changed to weekly retention meetings once full recruitment was achieved, and these were crucial to ensuring continued participation especially of the untreated control group, for the success of the SONIA 2 study. Although travel, accommodation and food expenses for participants were paid from the EC grant, problems were experienced in hospital administration systems for prompt payment of minor incidental expenses to participants which was eventually solved. There were 13 partners including two AKU patient societies in this large consortium and the weekly telemeetings ensured effective communication; there were also seven face-to-face meetings during DevelopAKUre to ensure the project progressed smoothly (Table 2).

**Table 2:** Barriers and solutions during the conduct of study

Barrier	How addressed
Fixed time for completion of study	Focussed and organised consortium; effective and rapid communication with funder European Commission
Lack of recruitment from UK	Recruited from outside UK
Travel for disabled patients	Patient society support and engagement; motivated patients
Language issues for overseas patients	Interpreters used
Complex protocol and extensive assessments	Assessments spread out over 5 days; all travel arrangements facilitated to minimise harship
Heterogeneous study sites	Careful harmonisation of processes and procedures; site initiation visits key
Continued patient participation	Patient Societies involved
Timely recruitment	Patient Societies involved. Weekly recruitment telemeetings
Retaining patients to complete study	Patient Societies involved. Weekly retention telemeetings
Efficient and timely completion	Weekly project board telemeetings Face to face yearly meetings

## Post-study considerations

The SONIA 2 study end in February 2019, data analysis was completed and the main publication setting out the results of the study was published in 2020 [18], while at the same time a compilation of documents were sent to the European Medicines Agency for label extension of NIT to cover its use in adults with AKU, also achieved in 2020 [19]. Further dissemination activities continue with over 20 articles published so far. Sobi, RLUH and AKU Society (UK) have assisted with national HTA assessments of NIT use in adults with AKU, often requiring several meetings with the national health regulatory authorities to ensure patient access to the study drug (Table 3).

**Table 3:** Barriers and solutions after completion of study

Barrier	How addressed
Timely data analysis	Efficient CRO and statistical support
Assessing study results	Seasoned experts from Sobi and efficient consortium including clinical experts
Dissemination	High profile publication prepared within 6 months of completion of data analysis
Regulatory approvals	Sobi undertook production of documents and communications with the European Medicines Agency
Further dissemination	More than 10 publications have already ensued from study
Patient access to study drug nitisinone	Sobi, Lead coordinator and AKU Society UK have achieved near Pan-European National HTA approvals of study drug

## Conclusions

Carrying out a successful clinical trial in very rare diseases is challenging. It requires careful planning and trans-national coordination. Regular meetings among all consortium partners ensured rapid problem identification and solving. Despite modest funding, much can be achieved in terms of delivering impactful and meaningful studies. Building a successful consortium used to working together collaboratively is crucial. Patients and their representatives have much to offer in ensuring successful recruitment and rentention [20]. The participation of a pharmaceutical partner as well as an effective consortium are crucial to success. Patient engagement with the programme by ensuring full participation is critical to success. Support from regulators in terms of facilitating clinical studies through realistic clinical trial outcomes is critical to success. We laud the European Commission for its focus on finding solutions for rare diseases through its funding programs without which progress will not come readily for many.

## Data Availability

Not applicable.

## Abbreviations

AKU: Alkaptonuria
EC: European Commission
HGA: Homogentisic acid
HRRP: *p*-Hydroxyphenyl-pyruvate dioxygenase
HT-1: Hereditary tyrosinaemia 1
HTA: Health technology assessment
NAC: National AKU Centre
NIH: National Institutes of Health
NIT: Nitisinone
RLUH: Royal Liverpool University Hospital
Sobi: Swedish Orphan Biovitrum
SONIA 1 and SONIA 2: Suitability of Nitisinone in Alkaptonuria 1 and 2
